# Looking Beyond Pneumonia and Asthma in India: An Interesting Case of Churg-Strauss Syndrome

**DOI:** 10.7759/cureus.66416

**Published:** 2024-08-08

**Authors:** Ashok Kumar, Manish Gaba, Naveen Kumar, Ashok Kumar

**Affiliations:** 1 Internal Medicine, Max Smart Super Specialty Hospital, Saket, New Delhi, IND; 2 Internal Medicine, Max Smart Super Specialty Hospital, Saket, Delhi, IND

**Keywords:** antibiotics, organism, bacteria, viruses, community acquired pneumonia, associated factors of asthma, eosinophilic granulomatosis with polyangiitis (egpa)

## Abstract

Churg-Strauss syndrome is a rare multisystem disorder characterized by asthma, eosinophilia, and vasculitis. The patient presented with prolonged fever, cough with blood-stained sputum, weight loss, pain in the abdomen, and a subsequent onset of hoarseness of voice. A history of asthma, left-side vocal cord paralysis, eosinophilia, nodular opacities on radiography, and eosinophilic duodenitis on biopsy led to a diagnosis of Churg-Strauss syndrome. The patient’s condition improved on treatment with steroids. This is an interesting case and presents an opportunity to learn about Churg-Strauss syndrome.

## Introduction

Churg-Strauss syndrome (CSS), also known as eosinophilic granulomatosis with polyangiitis (EGPA), is an autoimmune condition characterized by asthma, eosinophilia, extravascular granuloma formation, and vasculitic involvement of multiple organ systems [1]. The annual incidence of this disease is 1-3 per million [1]. It is 1.2 times more commonly seen in females [1]. Vasculitis caused by the disease mainly affects small- and medium-sized vessels. The pathologic findings consist of the infiltration of tissues with eosinophils. The disease predominantly involves the lungs but can affect any part of the body. Here we report a case of bronchial asthma presenting with fever, cough, and weight loss.

## Case presentation

A female in her 20s was admitted with complaints of fever up to 100° F for one month associated with generalized body aches. The patient also gave a history of productive cough which was occasionally blood-tinged. Two weeks prior to admission, she developed severe upper abdominal pain which was non-radiating, dull aching type, and worsening on food intake. Since the onset of pain abdomen, her fever increased, touching up to 103F. The patient subsequently presented to the emergency with rapidly worsening shortness of breath for 1-day duration. The patient also gave a history of weight loss of 7-8 kg over this period. She had a background history of bronchial asthma, for which she was using inhalers for 10 years with intermittent steroid use to manage exacerbations. On examination, she had a heart rate of 110 beats per min, blood pressure of 110/70 mmHg, was tachypnoeic with a respiratory rate of 24 breaths per min, and an oxygen saturation of 85% on room air going up to 94% on oxygen support of 5 liter/minute via face mask. Her chest examination on auscultation revealed bilateral crepitation and rhonchi in all lung fields. The upper abdomen was tender in the epigastric region on palpation. Her neurological examination was normal and cardiovascular examination revealed tachycardia.

Investigations

The patient’s blood investigation, biochemistry, and urine analysis are mentioned in (Table 1). The complete blood counts revealed hemoglobin of 9.5 gm/dl, total leucocyte count of 38,700/mL, differential count of neutrophil 32%, lymphocyte 7%, monocyte 2%, eosinophil 56%, and platelet was 7,13,000/mL. This was suggestive of anemia with leucocytosis and eosinophilia. She had thrombocytosis which could be attributed to increased bone marrow turnover. Her absolute eosinophil count was 20,200/mL, peripheral smear revealed mild anisocytosis with eosinophilia and erythrocyte sedimentation rate (ESR) was 80 mm/hour. Her biochemistry, kidney function tests, liver function tests, and urine analysis were normal.

**Table 1:** Laboratory investigation ESR: erythrocyte sedimentation rate; SGOT: serum glutamic oxaloacetic transaminase; SGPT: serum glutamate pyruvate transaminase; ALP: alanine aminotransferase; GGT: gamma-glutamyl transpeptidase

Investigation	Values	Reference range
Hemoglobin (g/dL)	9.5	13-17
Total leucocyte count (cell/cum)	38,700	4-10
Platelet(cell/cumm)	7,13,000	150-410
Differential leucocyte count	Neutrophil 32%,lymphocyte 7%, monocyte 2%, eosinophil 56%	
Absolute eosinophil count(cells/cumm)	20,200	30-350
ESR (mm/hour)	80	<20
Creatinine (mg/dL)	0.8	0.9-1.3
Sodium (mmol/L)	135	136-146
Potassium (mmol/L)	4.0	3.5-5.1
Calcium (mg/dL)	9.6	8.8-10.2
SGOT (IU/L)	20	15-41
SGPT (IU/L)	30	17-63
ALP (IU/L)	88	32-91
GGT (IU/L)	40	7-50
Albumin (g/dL)	4	3.5-5
Urine analysis	0-1 wbc/hpf, 0 rbc/hpf, protein-neg, glucose-neg	

**Figure 1:**
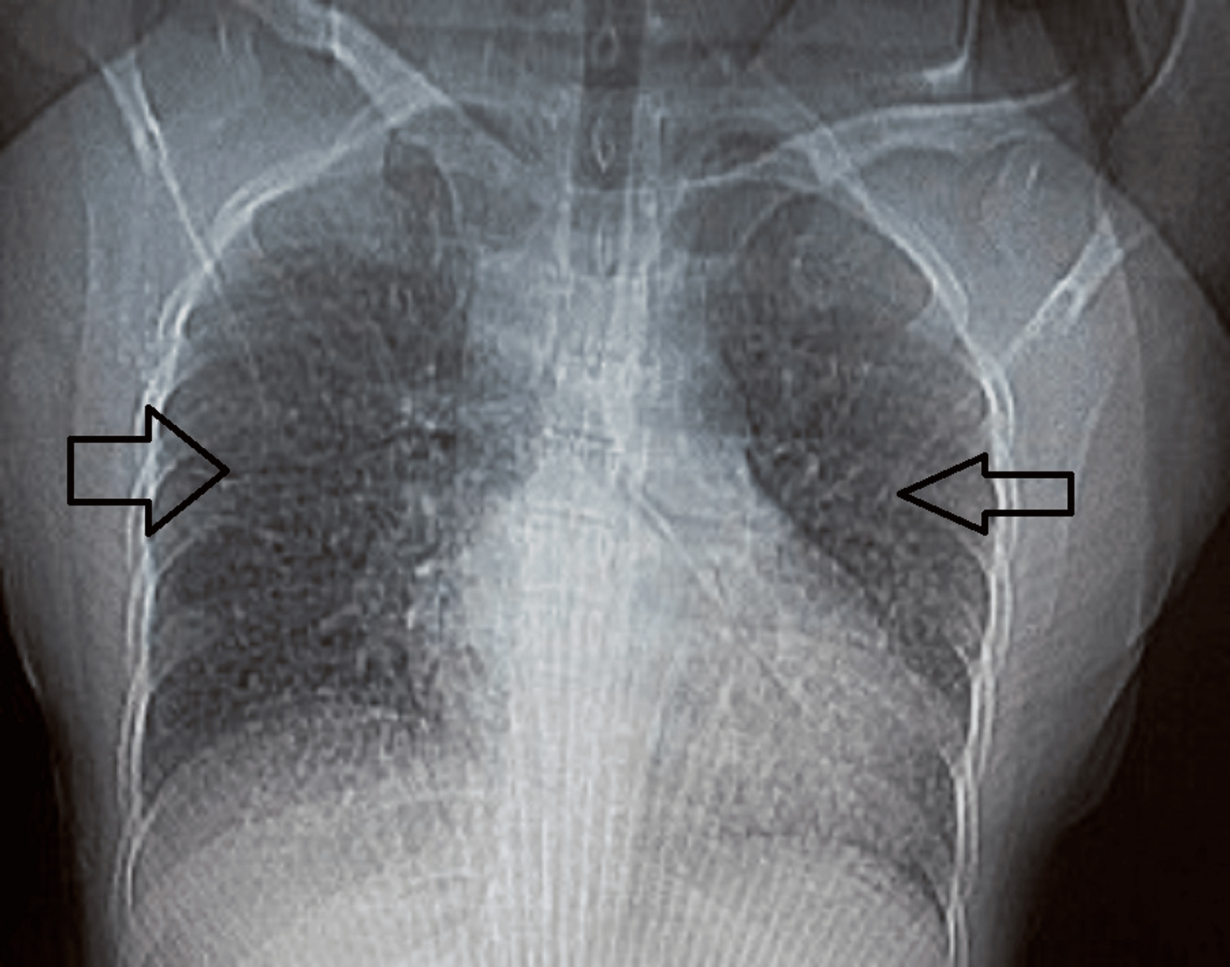
Chest X-ray showing bilateral nodular infiltrate in both lung fields.

**Figure 2:**
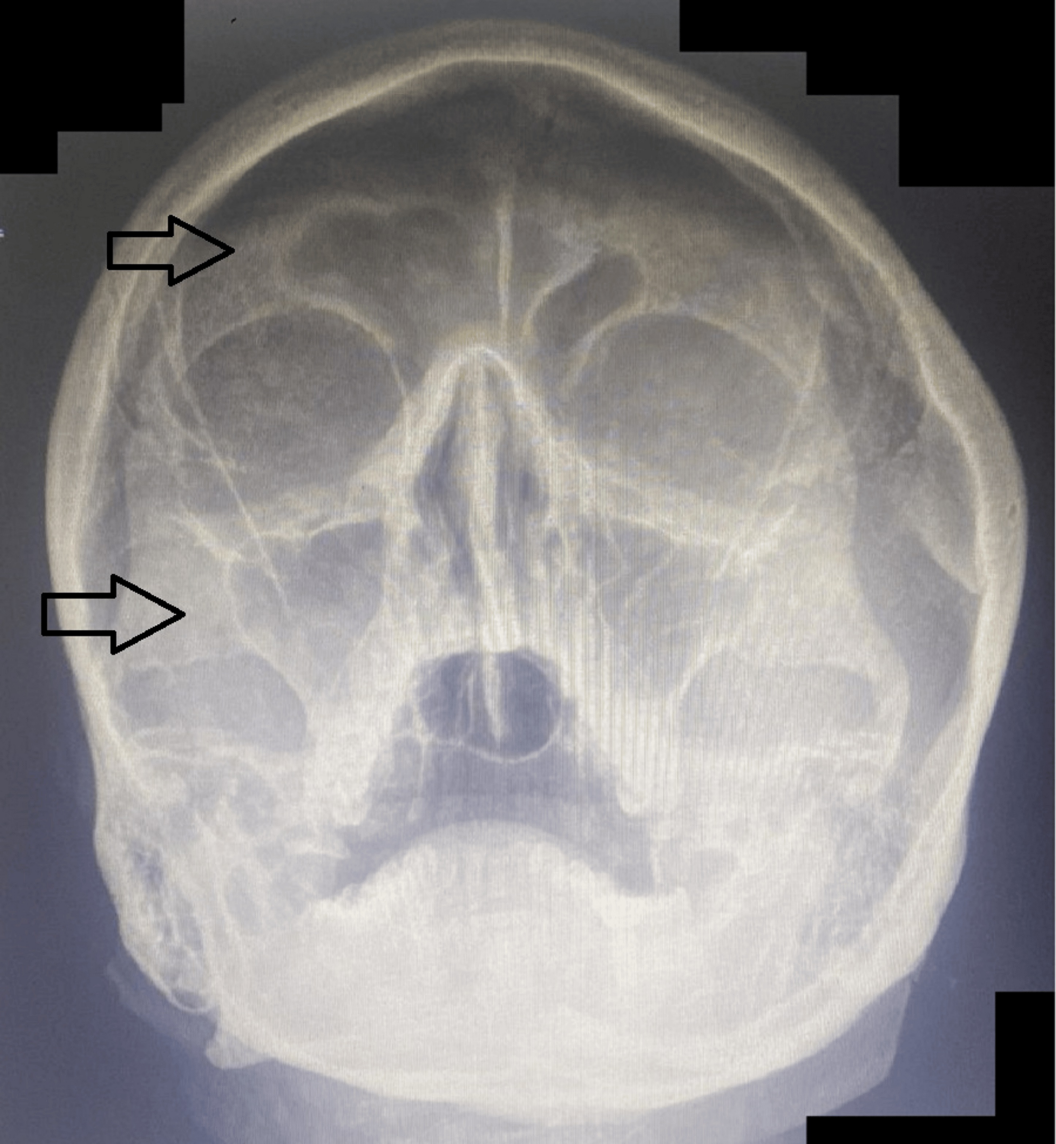
Radiograph of paranasal sinus showing bilateral maxillary sinus haziness and right frontal sinus haziness suggestive of sinusitis

The chest X-ray (Figure 1) revealed bilateral nodular opacities in both lung fields. The X-ray of the paranasal sinus had maxillary and frontal sinusitis (Figure 2).

The ECG showed sinus tachycardia and transthoracic echocardiography was normal. Her troponin-I, CK-MB, and NT-pro-BNP were normal. Her blood and urine cultures were sterile. The IgE level was very high (23459 IU/mL) indicative of an allergic phenomenon or a parasitic infection. The patient’s autoimmune profile was negative (Table 2).

**Table 2:** Autoimmune profile MPO: myeloperoxidase; GBM: anti-glomerular basement membrane

ANA	Negative
Ds-DNA	Negative
Nucleosome	Negative
Histone	Negative
SmD1	Negative
PCNA	Negative
PO	Negative
SSA/Ro60	Negative
SSA/Ro52	Negative
SSB/La	Negative
CENP-B	Negative
Scl-70	Negative
U1-snRNP	Negative
AMA-M2	Negative
Jo-1	Negative
PM-Scl	Negative
Mi2	Negative
Ku	Negative
MPO	Negative
GBM	Negative

**Figure 3:**
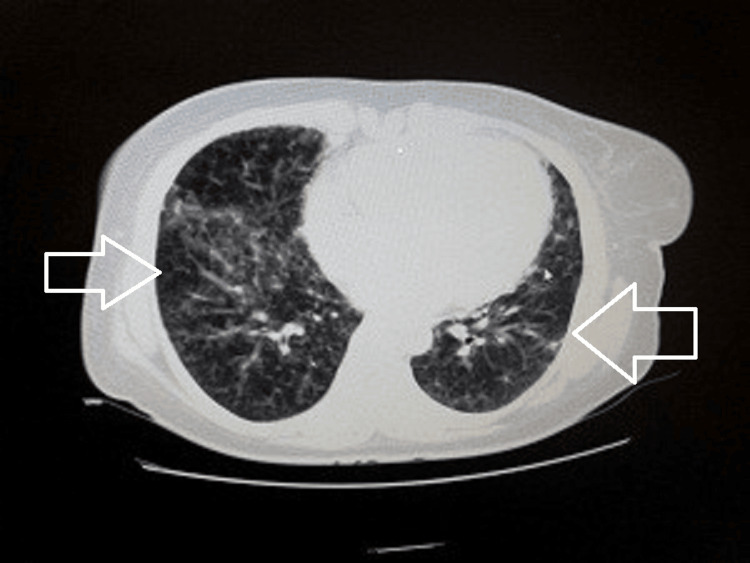
Multiple nodular opacities in bilateral lung fields involving upper and lower lobes.

**Figure 4:**
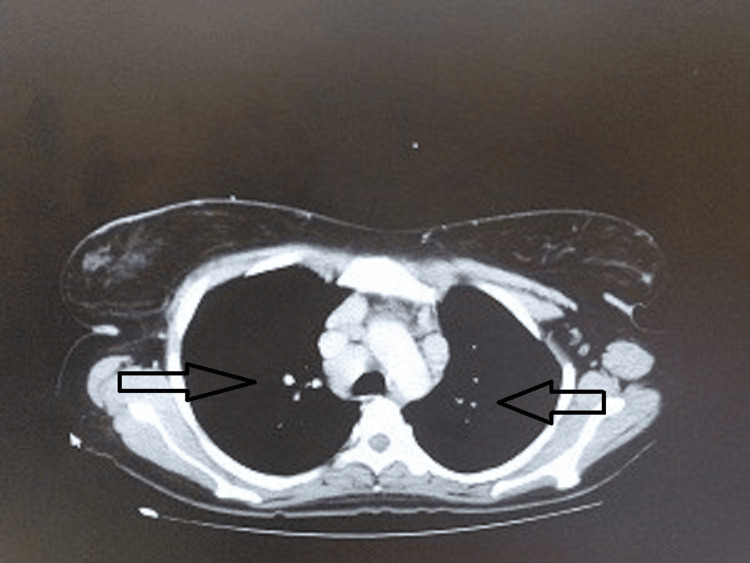
Multiple sub-centimetric lymph nodes seen in the mediastinal, pre-tracheal, para-tracheal, and hilar region.

Her sputum investigations showed no acid-fast bacilli, and the PCR for tuberculosis was negative. Her sputum culture grew *Escherichia coli*. This is an uncommon cause of pneumonia. A contrast-enhanced computed tomography (CT) chest was done which showed multiple nodular opacities in bilateral lung fields (Figure 3) involving upper and lower lobes and multiple sub-centimetric lymph nodes seen in the mediastinal, pre-tracheal, para-tracheal, and hilar regions (Figure 4).

**Figure 5:**
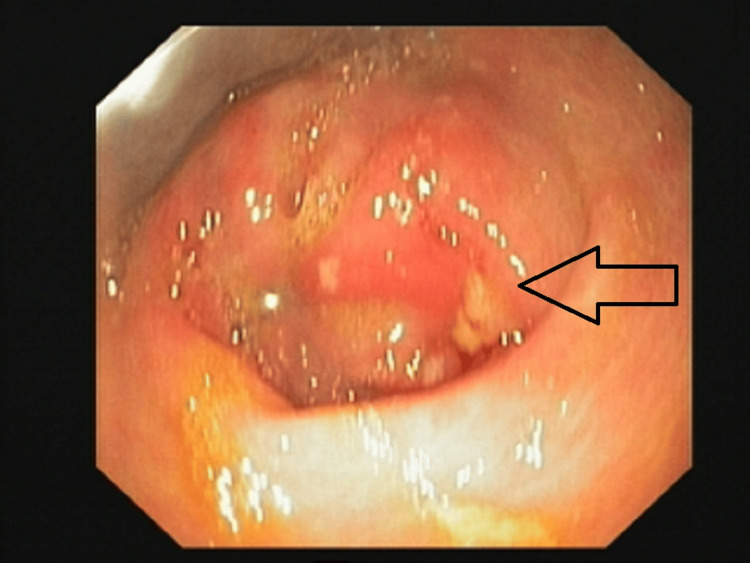
Upper GI endoscopy showing erythema with pre-pyloric ulcer

**Figure 6:**
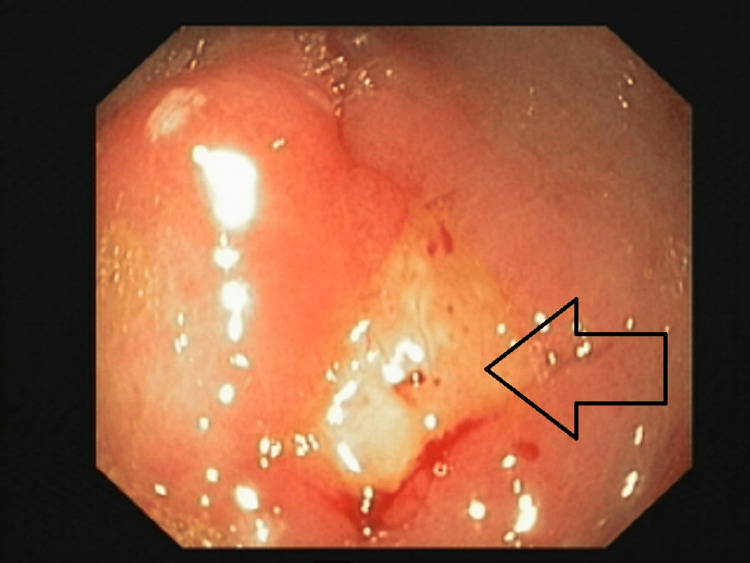
Upper GI endoscopy showing punctate white lesion over duodenal fold with elevated plaque

In view of respiratory failure, she was kept in the critical care unit and was managed with non-invasive ventilator support and oxygen support. As she had severe abdominal pain in the background of *E. coli*-associated pneumonia, a gastroenterology opinion was taken, and upper GI endoscopy was done which revealed pre-pyloric ulcers and punctate white lesions over duodenal folds (Figures 5-6). This would explain the *E. coli* pneumonia in the background of pyloric ulcers likely due to hematogenous dissemination.

**Figure 7:**
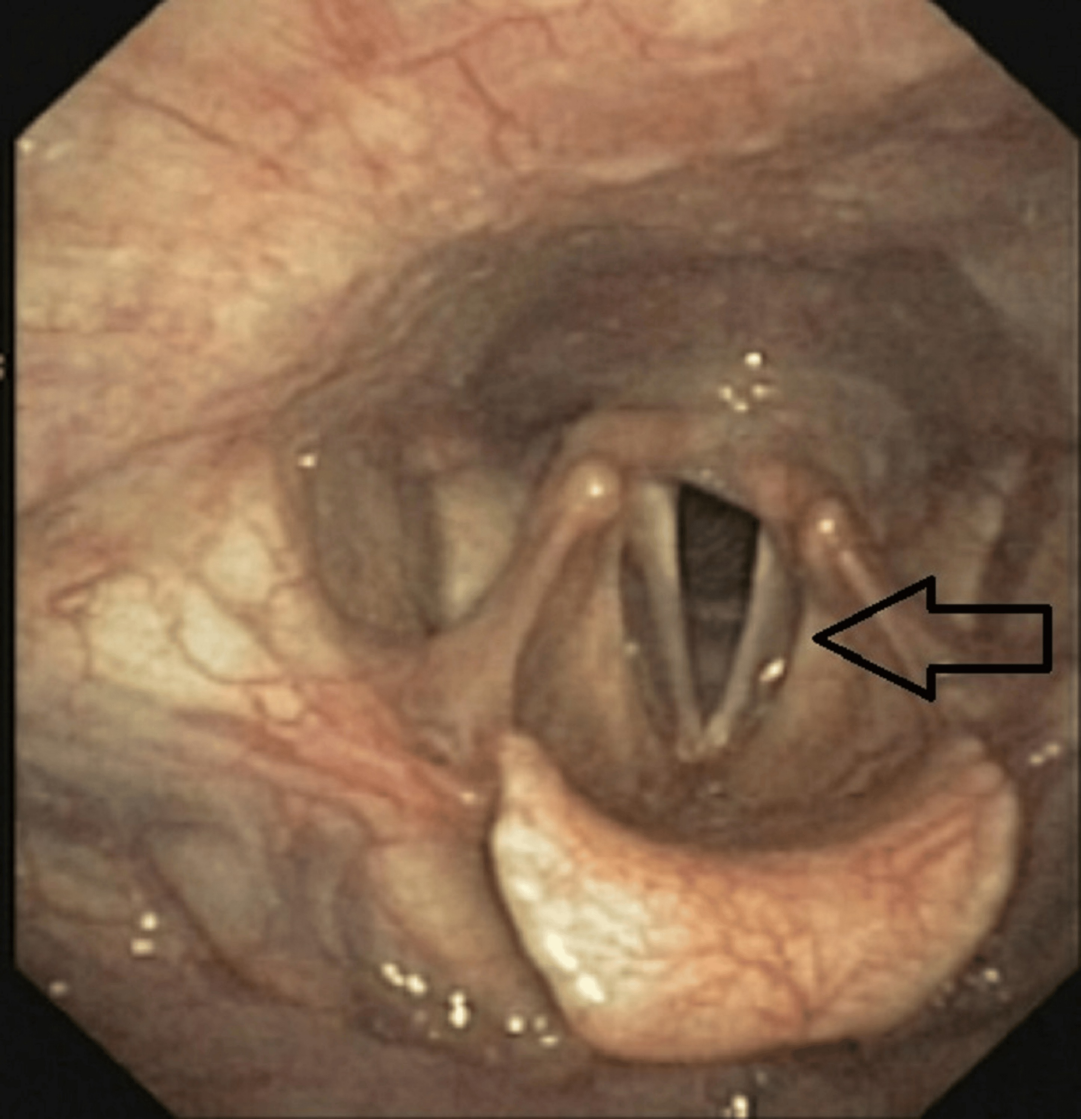
Direct laryngoscopy showing left vocal cord palsy.

A duodenal biopsy was taken which revealed eosinophilic duodenitis, thus confirming systemic infiltrative eosinophilic disease. The patient subsequently developed hoarseness of voice. An ENT opinion was taken and direct laryngoscopy was done which had an immobile left vocal cord with a phonatory gap in adduction (Figure 7). This was suggestive of neurological involvement seen in eosinophilic infiltrative diseases.

A hematology opinion was taken. A bone marrow biopsy was suggested to confirm systemic eosinophilic infiltrative illness and to rule out any eosinophilic leukemia. This revealed eosinophilic infiltrates but had no features of malignancy or granuloma. The patient’s systemic features (fever, anorexia, weight loss) along with respiratory, abdominal, and neurological findings in a background of bronchial asthma were suggestive of CSS with pneumonia.

Differential diagnosis

The patient had a background of asthma and now came with complaints of fever and cough. Our initial impression was worsening asthma secondary to bacterial pneumonia. However, with a long-standing history of symptoms associated with significant weight loss, we considered other possibilities. The patient was not improving on empiric antibiotics. Pulmonary Koch’s was considered as a differential. It is highly prevalent in India. In view of high eosinophil and background of asthma, we evaluated for CSS. Our gastroenterology team performed an upper GI endoscopy and the biopsy taken from GI ulcers revealed eosinophilic duodenitis. We consulted the hematologist and a bone marrow aspiration and biopsy were advised, which revealed eosinophilic infiltrates. All these findings lead us to a diagnosis of CSS with pneumonia.

Treatment

Keeping an initial differential diagnosis of exacerbation of bronchial asthma with a suspected lower respiratory tract infection with type 1 respiratory failure, she was initiated on IV antibiotics, oxygen support along with other supportive care. Parasitic infestations are very common in India and in the background of eosinophilia and elevated IgE levels, the patient was dewormed with Albendazole. Her subsequent investigation findings of nodular infiltrates on chest X-ray and CT chest were suggestive of a diagnosis of CSS. The patient was started on steroids: IV methylprednisolone in a dose of 15 mg/kg/day. The patient responded well with steroids. Her oxygen requirement decreased and she was weaned off oxygen support. In view of good response, the patient was kept on steroids, and it was decided in a multi-disciplinary team meeting to re-evaluate the patient on follow-up.

The patient improved symptomatically and she was discharged on oral steroids. The patient had a remarkable improvement in her symptoms on follow-up with normal eosinophil counts and IgE levels. The patient was advised to be started on azathioprine to maintain remission. This was selected as she was a female patient in her 20s. The patient has not come for subsequent follow-ups.

## Discussion

The patients with CSS present with non-specific symptoms like fever and weight loss. The main findings of the disease are recurrent attacks of asthma and pulmonary infiltrates on chest radiography. Neurological involvement is the second most common presentation with mononeuritis multiplex seen in 72% of cases [1]. These patients often have a past history of allergic rhinitis and sinusitis. Skin lesions like purpura, cutaneous, and subcutaneous nodules are also seen. Heart disease with myocarditis, pericarditis, endocarditis, and coronary vasculitis is seen in 14% of patients and is a leading cause of mortality. Gastrointestinal tract symptoms consist of pain abdomen usually due to peritonitis, ulceration, and perforation. Renal involvement is less common. Subsequently, vasculitis ensues which can lead to life-threatening complications [1].

**Table 3:** The American College of Rheumatology 1990 criteria

The diagnosis of CSS is based on The American College of Rheumatology 1990 criteria [2]
Asthma
Eosinophils greater than 10% of a differential white blood cell count
Presence of mononeuropathy or polyneuropathy
Unfixed pulmonary infiltrates
Presence of paranasal sinus abnormalities
Histological evidence of extravascular eosinophils

The patient is diagnosed as a case of CSS if at least four of these six criteria are positive (Table 3). The presence of any four or more of the six criteria yields a sensitivity of 85% and a specificity of 99.7%.

The French Vasculitis Study Group has developed a five-point system (five-factor score) that predicts the risk of death in CSS using clinical presentations (Table 4) [3].

**Table 4:** Risk of death in Churg-Strauss syndrome using clinical presentations-French vasculitis study group

Risk of death in Churg-Strauss syndrome using clinical presentations-French vasculitis study group
Reduced renal function (creatinine >1.58 mg/dL or 140 μmol/L)
Proteinuria (>1 g/24h)
Gastrointestinal hemorrhage, infarction, or pancreatitis
Involvement of the central nervous system
Cardiomyopathy

The patients with none of these clinical features have a five-year mortality rate of 11.9%. The presence of one factor indicates severe disease, with a five-year mortality rate of 26%. Two or more of these findings indicate very severe disease with a 46% five-year mortality rate [3].

The prognosis of untreated disease is very poor with a 5-year survival of 25%, which improves to 72% in patients who receive treatment [1]. Glucocorticoids constitute the main line of management. Methylprednisolone is used in moderate or severe cases at a dose of 15 mg/kg/day. After the initial vasculitis settles, the dose of steroids is tapered and the patient can be started on prednisolone. Many patients may require low-dose prednisolone for persistent asthma. Individuals with cardiac involvement should be treated with a combination of glucocorticoids and cyclophosphamide followed by azathioprine or methotrexate. mepolizumab has been studied in randomized trials and has been shown to be effective [4]. Rituximab has also been used for management of CSS [5]. CSS cases must be followed up very closely. The patient’s clinical status, ESR, and eosinophilia should be monitored.

Our patient was initially suspected to be a case of tuberculosis. Tuberculosis is a very common disease entity in India [6]. Tuberculosis can present with a wide variety of symptoms. It is an important differential diagnosis for patients presenting with a history of long-standing low-grade fever, weight loss, and cough in India. Confirming the diagnosis can be difficult despite detailed investigation. They are often treated empirically with anti-tubercular drugs based on suspicion. However, this approach could be counter-productive as uncommon entities could be missed. A high index of suspicion should always be kept for uncommon conditions. An interesting case was reported by Zonzin et al. a female patient in Brazil who was diagnosed and treated empirically as a case of tuberculosis [7]. The patient deteriorated and eventually landed in the ICU. She was subsequently diagnosed as a case of CSS. It was mentioned by the author that this patient did not have a history of asthma which led to difficulty in making the diagnosis [7].

An interesting case report by Mekic et al. has several parallels with our case [8]. Their patient was admitted for a cholecystectomy. His post-operative period was uneventful and he was discharged. The patient developed a fever 10 days after his discharge. In the workup, he was diagnosed with pneumonia and wound site infections which were treated with antibiotics. The blood work showed high eosinophil counts. Gall bladder biopsy revealed mixed infiltrate with a predominance of eosinophil granulocytes and numerous congested vessels. He was suspected to be a case of Churg-Strauss syndrome and was started on steroids and after improvement clinically and was discharged. This patient subsequently came back as a case of acute heart failure. He was readmitted and treated with steroids and his condition improved over the course of 7 days. This case highlights the importance of clinical suspicion and performing a complete workup.

CSS shows a very good response to steroids. Cardiac complications are life-threatening events in the case of CSS and must be promptly managed. In a case reported by Amelotti et al., the patient had eosinophilic myocarditis which improved on therapy with corticosteroids [9].

The biopsy from the pyloric ulcer along with the findings of vocal cord paralysis guided us in reaching our diagnosis. An interesting case report by Mazzantini et al. showed that vocal cord palsy can be a manifestation of CSS [10]. Our case highlights the importance of a diagnostic workup which leads to the recognition of rare but treatable diseases.

## Conclusions

CSS is a rare multisystem disease entity. A history of asthma in a patient presenting with symptoms involving multiple systems must always prompt evaluation of CSS. India has a high incidence of tuberculosis which can present as a multisystem disease. A high index of suspicion is important to look for other possible diagnoses. Parasitic infestation is also an important cause that should be treated especially in India. Our case emphasizes the importance of a strong clinical suspicion, which should be followed by a syndromic approach based on a complete workup.
